# Supplementary material for: Metabolic Potential for Reductive Acetogenesis and a Novel Energy-Converting [NiFe] Hydrogenase in Bathyarchaeia From Termite Guts – A Genome-Centric Analysis
Source: Front Microbiol. 2021 Feb 3;11:635786. doi: 10.3389/fmicb.2020.635786 (PMC7886697; doi:10.3389/fmicb.2020.635786)
Supplement: Supplementary Figure 2 — Genome-based phylogeny of termite gut Bathyarchaeia illustrating the relationship of lineages TB1 and TB2 to other MAGs in the Bathy-6 subgroup. MAGs mentioned in the text are marked in bold. The maximum-likelihood tree was inferred from a concatenated alignment of 43 proteins using the LG+F+I+G4 model and rooted with selected Crenarchaeota and Euryarchaeota as outgroup. The numbers in circles indicate the phylotypes discussed in the text (Table 1). MAGs included in the comparative analysis (Figure 3) are shown in bold. The tree was rooted other archaeal genomes as outgroup. The scale bar indicates 10-amino-acid substitutions per site. Node support values (SH-aLRT) are shown in blue. A simplified version of the tree is shown in Figure 1. [file Data_Sheet_2.pdf]

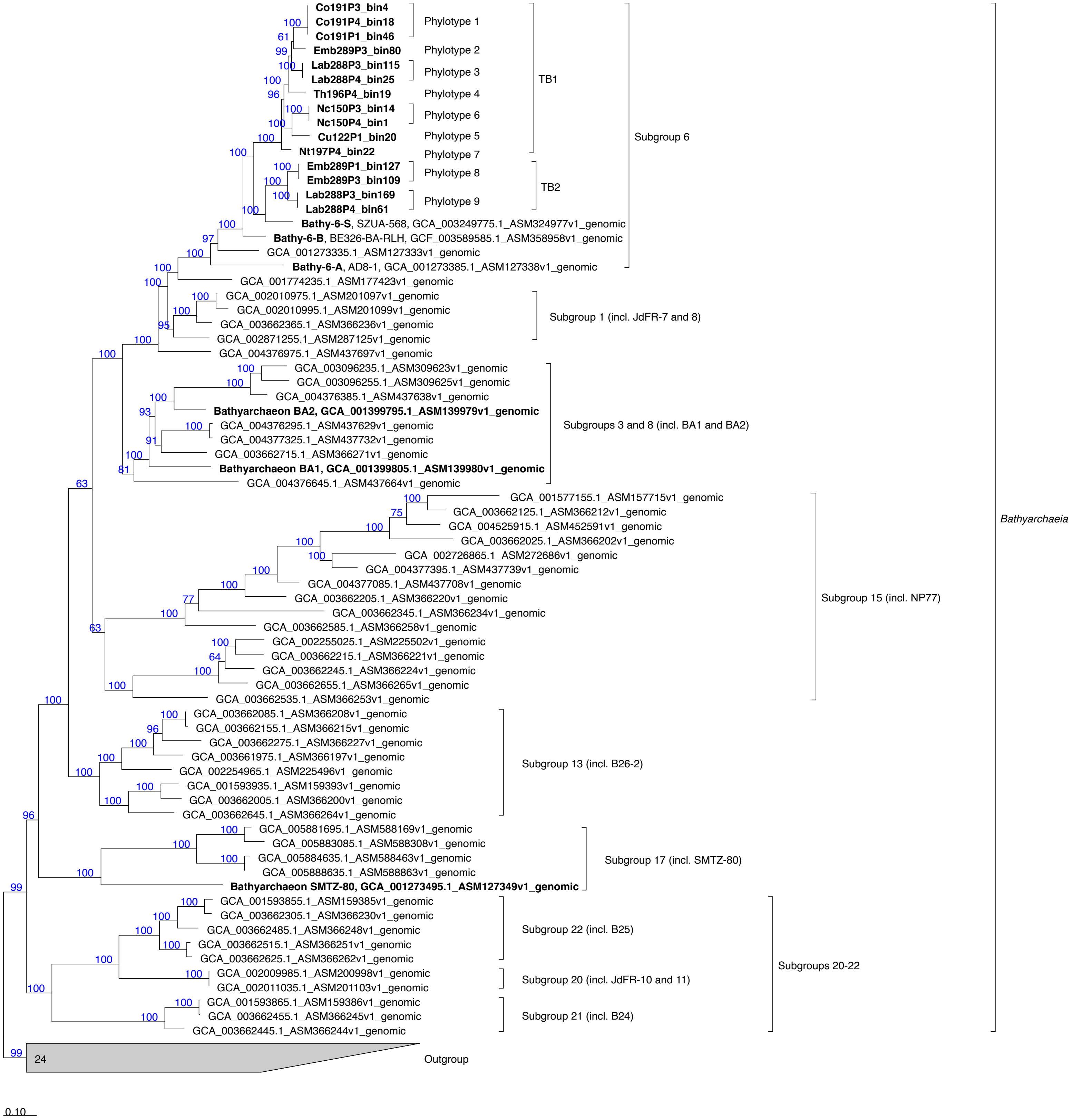

Supplementary Figure S2. Genome-based phylogeny of termite gut *Bathyarchaeia*. The maximum-likelihood tree was inferred from a concatenated alignment of 43 proteins using the LG+F+I+G4 model. The numbers in circles indicate the phylotypes discussed in the text (Table 1). MAGs included in the comparative analysis (Figure 3) are shown in bold. The tree was rooted other archaeal genomes as outgroup. The scale bar indicates 0.1 amino acid substitutions per site. Node support values (SH-aLRT) are displayed at each branch. A simplified version of the tree is shown in Figure 1.
