# Supplementary material for: Metabolic Potential for Reductive Acetogenesis and a Novel Energy-Converting [NiFe] Hydrogenase in Bathyarchaeia From Termite Guts – A Genome-Centric Analysis
Source: Front Microbiol. 2021 Feb 3;11:635786. doi: 10.3389/fmicb.2020.635786 (PMC7886697; doi:10.3389/fmicb.2020.635786)
Supplement: Supplementary Figure 3 — 16S rRNA-based phylogeny of subgroup Bathy-6, indicating the placement of the sequences from termite guts among those obtained from other environments. The maximum-likelihood tree is based on a curated alignment (1,424 positions) of all sequences in the SILVA database and their homologs retrieved from the bathyarchaeal MAGs (in bold) and the low-quality bins obtained from the termite gut metagenomes (Hervé et al., 2020). The tree was rooted using members of Bathy-5 as outgroup. The scale bars indicate 0.05 nucleotide substitutions per site. Node support values (SH-aLRT) are shown in blue. Branches marked with dashed lines indicate shorter sequences that were added using the ARB parsimony tool. A simplified version of the tree is shown in Figure 2. [file Data_Sheet_3.pdf]

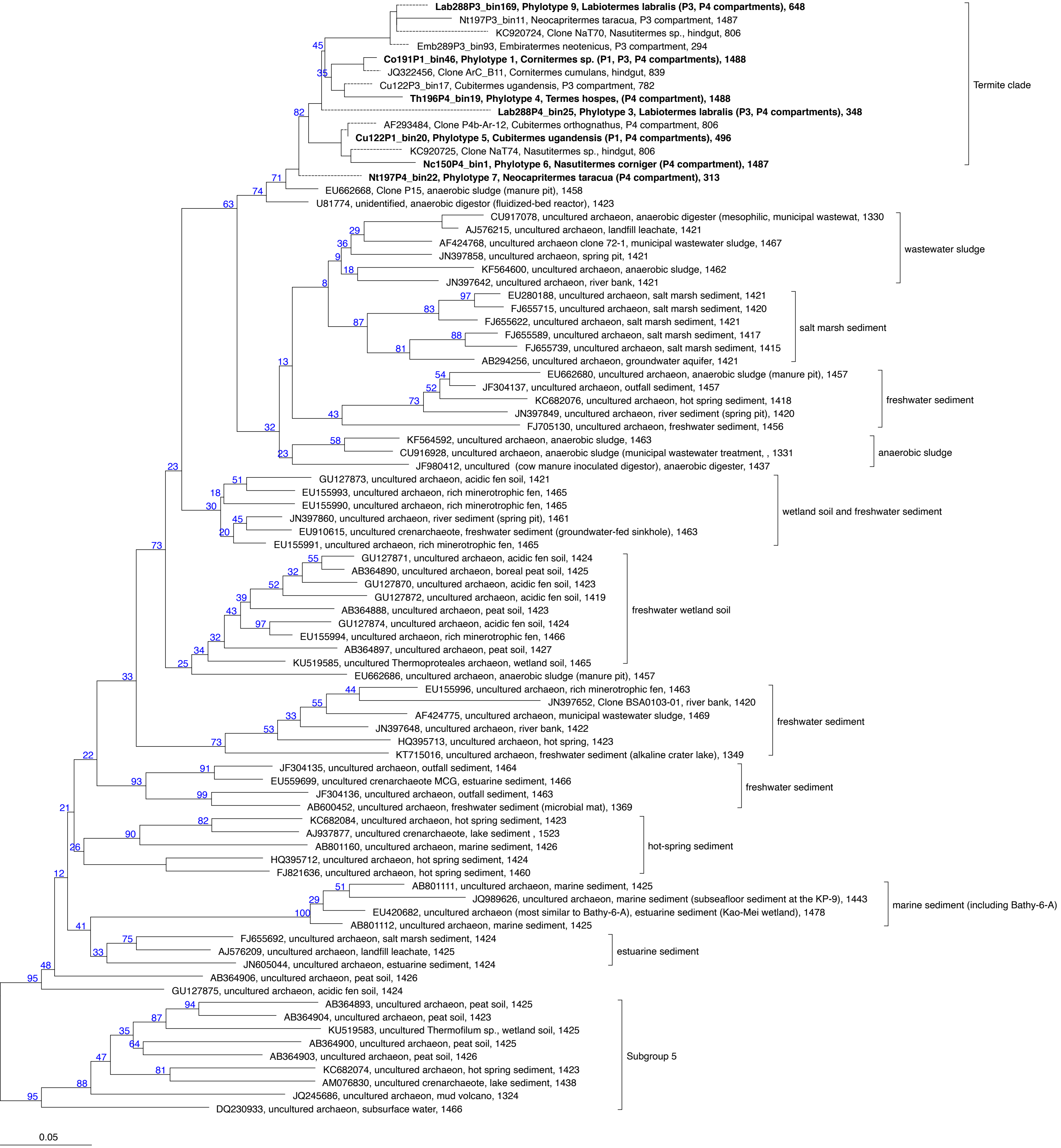

Supplementary Figure S3. 16S rRNA gene tree of Bathy-6, indicating the placement of the sequences from termite guts (in bold) among those obtained from other environments. Tree topology was inferred by ML analysis of an unambiguous alignment of the 16S rRNA gene sequences longer than 1300 bp retrieved from the MAGs and also all low-quality bins from the termite gut metagenomes that were classified as Bathyarchaeia (Hervé et al., 2020) in the framework of the SILVA database. The tree was rooted using members of Bathy-5 as outgroup. The scale bars indicate 0.05 nucleotide substitutions per site. Node support values (SH-aLRT) are displayed at each branch. A simplified version of the tree is shown in Figure 2.
