# Supplementary material for: Metabolic Potential for Reductive Acetogenesis and a Novel Energy-Converting [NiFe] Hydrogenase in Bathyarchaeia From Termite Guts – A Genome-Centric Analysis
Source: Front Microbiol. 2021 Feb 3;11:635786. doi: 10.3389/fmicb.2020.635786 (PMC7886697; doi:10.3389/fmicb.2020.635786)
Supplement: Supplementary Figure 4 — The methyltransferase-associated corrinoid protein (CoP) of Bathy-6 and its homologs. (A) The canonical methyltransferase system of bacteria and archaea. (B) Gene neighborhood of the CoP gene of Bathy-6 and selected homologs [for accession numbers, see panel (C)]. Colors indicate the presumed functions of the respective gene products (A). Unrooted phylogenetic trees of the methyltransferase-associated CoP genes (C) and the associated mtrH genes (D) of Bathy-6 and their closest relatives (deduced amino acid sequences). Genes that appear in (D) are shown in bold. Numbers are IMG/Mer gene IDs. The scale bar indicates 1.0-amino-acid substitution per site. Node support values (SH-aLRT) are shown in blue. [file Data_Sheet_4.PDF]

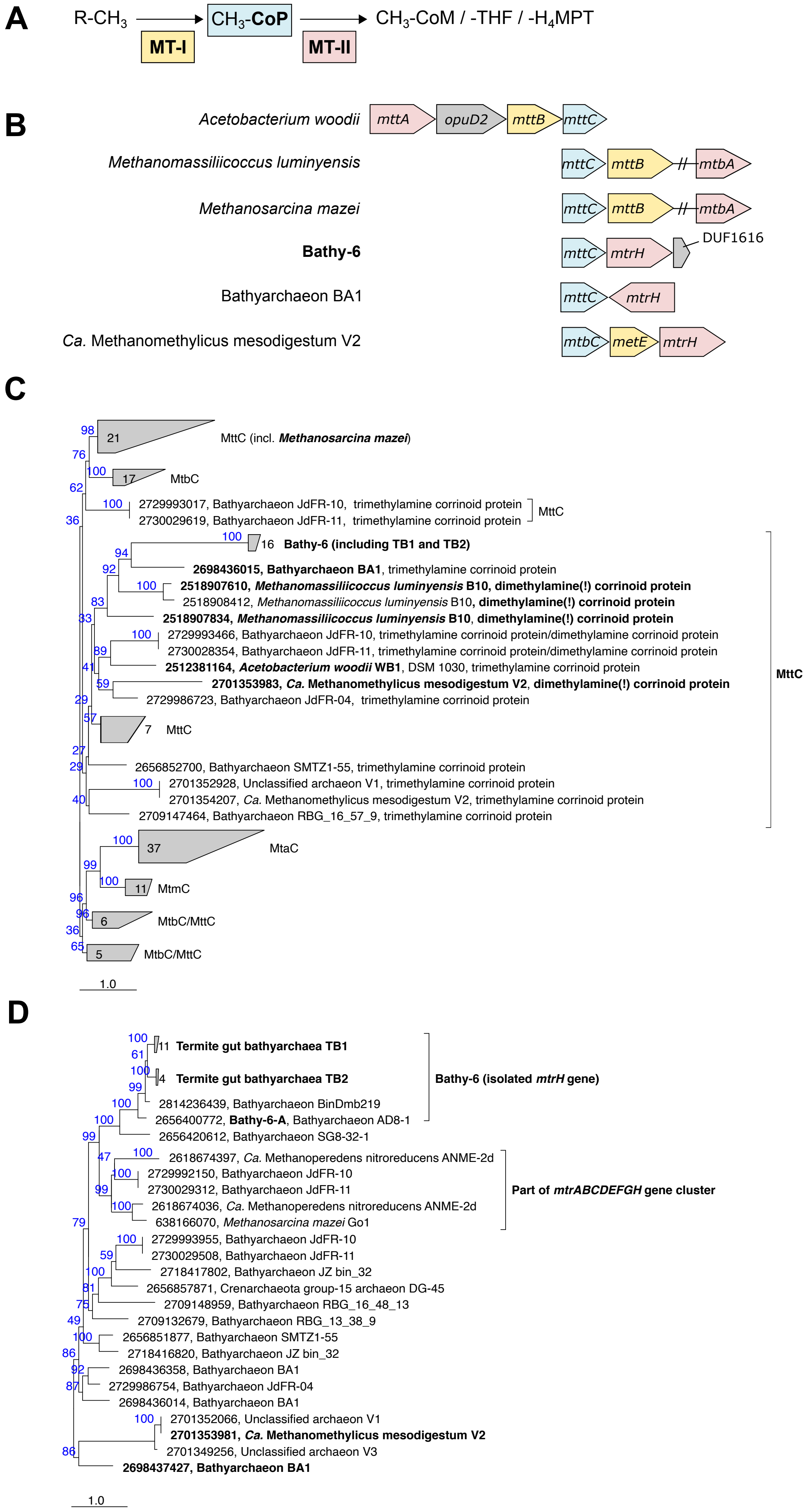

Supplementary Figure S4. The methyltransferase-associated corrinoid protein (CoP) of Bathy-6 and its homologs. (A) The canonical methyl transferase system of bacteria and archaea. (B) Gene neighborhood of the CoP gene of Bathy-6 and selected homologs (for accession numbers, see panel C). Colors indicate the presumed functions of the respective gene products (see panel A). Unrooted phylogenetic trees of the methyltransferase-associated CoP genes (C) and the associated mtrH genes (D) of Bathy-6 and their closest relatives (deduced amino acid sequences). Genes that appear in panel D are shown in bold. Numbers are IMG/Mer gene IDs. The scale bar indicates 1.0 amino acid substitution per site. Node support values (SH-aLRT) are shown in blue.
