# Supplementary material for: Metabolic Potential for Reductive Acetogenesis and a Novel Energy-Converting [NiFe] Hydrogenase in Bathyarchaeia From Termite Guts – A Genome-Centric Analysis
Source: Front Microbiol. 2021 Feb 3;11:635786. doi: 10.3389/fmicb.2020.635786 (PMC7886697; doi:10.3389/fmicb.2020.635786)
Supplement: Supplementary Figure 5 — Phylogenetic tree of the catalytic subunit of the Hox hydrogenase of Bathy-6 and its homologs among group 3 [NiFe] hydrogenases. The maximum-likelihood tree is based on deduced amino acid sequences and was rooted [NiFe] hydrogenase sequences of groups 1 and 2. The scale bar indicates 0.5 nucleotide substitutions per site. Node support values (SH-aLRT) are shown in blue. [file Data_Sheet_5.PDF]

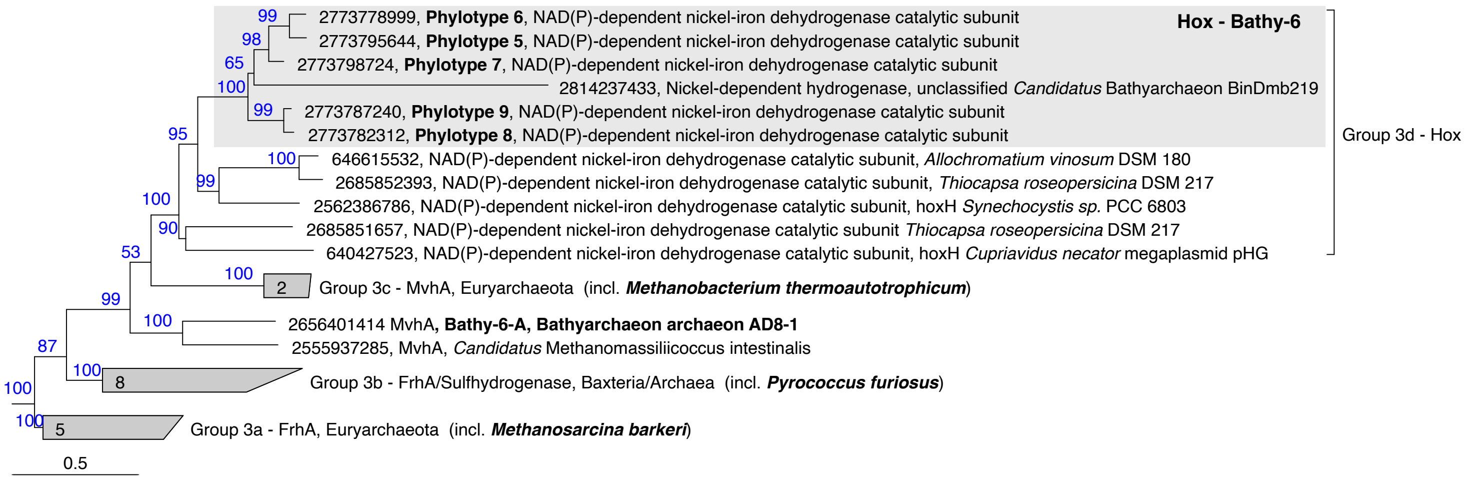

Supplementary Figure S5. Phylogeny of the catalytic subunit of the Hox hydrogenase of Bathy-6 and its homologs among Group 3 [NiFe] hydrogenases. The tree was rooted with groups 1 and 2 [NiFe] hydrogenase sequences. Tree topology was inferred by maximum-likelihood; the scale bar indicates 0.5 nucleotide substitutions per site. Node support values (SH-aLRT) are displayed at each branch.
